# Mechanisms, translational opportunities for diabetic polyneuropathy

**DOI:** 10.3389/fendo.2026.1899469

**Published:** 2026-07-23

**Authors:** Honyi Ong, Mingyang Hong, Douglas W. Zochodne

**Affiliations:** Division of Neurology, Department of Medicine, Neuroscience and Mental Health Institute, University of Alberta, Edmonton, AB, Canada

**Keywords:** axon regeneration, axonal degeneration, diabetes mellitus, diabetic polyneuropathy, sensory loss

## Abstract

Diabetic polyneuropathy (DPN) is a common complication of types 1 and 2 diabetes mellitus (DM). Peripheral sensory axons are early and primary targets of DPN, rendering sensory loss, pain and impaired quality of life. While several metabolic and other abnormalities, including abnormal insulin signaling and mitochondrial dysfunction contribute toward DPN, effective therapy to reverse or arrest the disorder is not currently available. DPN is a degenerative condition that involves retraction of distal sensory terminals and failure of regenerative repair. This review summarizes current mechanisms of DPN and potential new approaches that may offer translational opportunities for treatment. Many of these targets have yet to be explored in multiple DM models/paradigms and have not yet entered clinical trials. Nonetheless, specific regeneration pathways may offer a future role to encourage sensory axon resilience and regrowth.

## Introduction

Diabetic neuropathy is among the most common complications in type 1 and type 2 diabetes mellitus (T1DM & T2DM) (1). According to the International Diabetes Federation (IDF) in 2025, an estimated 589 million adults (11.1% of world population) aged 20–79 years worldwide are living with diabetes mellitus (DM). By 2050, the total number of adults living with DM is projected to rise to 853 million (13% of world population) (2). Diabetic polyneuropathy (DPN) is the most prevalent chronic complication of T1DM and T2DM, involving over half of all diabetic persons, significantly compromising patients’ quality of life and contributing to elevated morbidity and mortality rates (3). DPN develops within 10 years of the onset of diabetes in T1DM patients, while for T2DM patients, DPN may already be present at the time of diagnosis or preceding this, in the prediabetes stage (4). For example, in patients diagnosed with diabetes, it is estimated that over 20% already have DPN (5). DPN imposes a substantial economic burden on healthcare systems, patients and families (6).

The disorder is characterized by a degenerative symmetrical loss of distal axons, starting in the extremities, recognized as a ‘glove and stocking syndrome’, leading to sensory loss and neuropathic pain. Autonomic dysfunction and later motor dysfunction are also features, not discussed here. DPN is a leading cause of foot ulceration and lower limb amputations. Key pathogenic pathways or mechanisms identified include activation of the polyol pathway resulting in sorbitol accumulation, increased oxidative stress, mitochondrial dysfunction, accumulation of advanced glycation end products (AGEs) and perhaps microvascular impairment (7). While DPN can sometimes be prevented or delayed with careful glycemic control, established DPN is often irreversible (8). In work to date, interventions to reverse neuronal metabolic targeting include aldose reductase inhibitors, sodium-glucose cotransporter 2 (SGLT2) inhibitors, glucagon-like peptide-1 (GLP-1) receptor agonists or others. These have all had varying but not robust impacts on DPN (9–11). Current management strategies primarily focus on controlling hyperglycemia, alleviating symptoms of diabetes and controlling pain. Disease-modifying treatments capable of arresting or reversing progressive axon loss in DPN remain elusive.

## Subtypes of diabetic neuropathy

### Polyneuropathy

The most prevalent diabetic neuropathy is distal symmetric polyneuropathy (diabetic polyneuropathy; referred to here as DPN), accounting for approximately 75% of patients with diabetic neuropathy (12). Symptoms of DPN include ‘stocking and glove’ pattern numbness, pain, with weakness of distal extremities in more advanced disease. Approximately 10% to 30% patients diagnosed with DPN have neuropathic pain depending on the populations studied (13–18). DPN is associated with unrecognized injuries, ulcerations, and amputations, as individuals may not detect harmful stimuli, leading to increased mortality: 5-year mortality rates of patients with diabetic foot ulcers was 2.5 times higher than in patients without foot ulcers (19). According to a study conducted among Chinese and European persons, painful DPN may be particularly prevalent in patients diagnosed with diabetes of durations less than 10 years or alternatively longer, in those with DM of more than 15 years. Older age, female gender, and BMI ≥ 30 kg/m^2^ were also risk factors of painful diabetic neuropathy (PDN) (20). Early small fiber involvement is associated with burning pain, painful paraesthesiae, pinprick insensitivity, hyperalgesia, and allodynia. Large fiber involvement can cause numbness, loss of vibration and proprioception, paraethesiae, motor weakness, and imbalance with unintentional injury (1, 21, 22).

### Autonomic neuropathy

Autonomic neuropathy may develop with or independently of DPN. Its targets include cardiovascular, digestive, urinary, sexual, and sudomotor systems (23). Cardiovascular autonomic neuropathy (CAN) can be easily overlooked because it is asymptomatic in early stages (21). Subclinlical CAN may be identified by heart rate variability (HRV) analysis (24) whereas later symptoms include palpations, orthostatic hypotension (1) or cardiac congestive failure and sudden death (23). Esophageal dysfunction can include dysphagia and heartburn whereas gastric dysfunction can manifest with vomiting, abdominal pain, early satiety, and bloating. Lower GI tract related symptoms include constipation, diarrhea, and fecal incontinence (25). Finally, urogenital neuropathy impairs bladder storage, voiding and sexual function (26).

### Focal neuropathies

Mononeuropathy and radiculoplexopathy, may occur with or without DPN and present with local pain, weakness, and loss of sensation rather than symmetric neurological symptoms (27–30). Some focal neuropathies, such as carpal tunnel syndrome and ulnar neuropathy at the elbow are related to compression, but others such as oculomotor paralysis and lumbosacral plexopathy may have an ischemic etiology.

## Neuropathy in types 1 and 2 diabetes mellitus

The incidence and prevalence of DPN in T1DM and T2DM vary widely across different studies. The Rochester diabetic cohort study suggested that almost two thirds of persons with T1DM and a similar, albeit slightly lesser proportion of T2DM persons had neuropathy. Less than 20% had symptomatic neuropathy and a smaller proportion had advanced neuropathy impairing mobility, sensation and autonomic function (6% in T1DM, 1% in T2DM) (17). The DCCT/EDIC group suggested that at least 20% of T1DM patients developed DPN after 20 years (31). Approximately 10-15% persons with newly diagnosed T2DM may have DPN (32) and 50% after 10 years. From a structural perspective, some experimental T1DM models show early axonal swelling, later axonal atrophy, fiber loss, and node/paranodal apparatus abnormalities and the findings were more prominent than in rats with T2DM (33). In patient cohort studies, namely the Diabetes Control and Complications Trial, while rigorous glucose control treatment decreased retinopathy risk and neuropathy incidence by 76% and 60% respectively in T1DM (34) there were only modest (5%–9% reduction) or negligible impacts in T2DM patients (35–38). These findings all indicate different potential mechanisms between T1DM and T2DM development, to be discussed further below.

## Mechanisms of diabetic polyneuropathy

### Sensory impairment

DPN prominently targets sensory axons and this occurs earlier than motor axon damage. The reasons for this selectivity are uncertain (7, 39). The localization of motor neuron perikarya within the ventral horns of the spinal cord allows protection by the blood nerve barrier. In contrast, sensory neuron perikarya within dorsal root ganglia (DRGs) lack barrier protection and may be exposed to circulating toxins and metabolic changes of DM (40–43**).** Sensory neurons include those without myelination, that are classified as unmyelinated ‘C-fibers’. It is possible that these axons, including their distal terminals in the epidermis that lack Schwann cell (SC) support, are at greater risk of metabolic damage (40, 44). Subtle distal motor axon involvement with weakness may not be detected given the capacity of motor axons to collaterally reinnervate neighboring muscle fibers (45, 46**).** Sensory neurons also have higher excitability and faster inactivation requiring greater energy support from their mitochondria. For example, during topical stimulation to skin innervated by the saphenous nerve, elevated mitochondrial traffic in local sensory axons suggested a greater metabolic demand (47).

### Differing pathophysiology in types 1 and 2 DPN

In the type 1 BB/Wor-rat model, motor nerve conduction velocity declined by 30% after 5 weeks but changes were milder (12%) in type 2 BBZDR/Wor-rats (48). After two months of DM, sensory nerve conduction velocity declined by 14% in T1DM but not at all in T2DM in one study (49). Further, T1DM (STZ) had better glycemic responses and improved neuropathy compared to T2DM db/db mice when treated with insulin (see below (50)). Thus, T2DM may be considered as a disorder of “insulin resistance”, classically linked to impairment of its action in adipose and muscle tissues. However, resistance can also be identified in the trophic response to insulin of neurons (51). Insulin resistance has been linked to upregulation of c-Jun N-terminal kinase (JNK) activity, and inappropriate phosphorylation and dysfunction of insulin receptor substrate (IRS) (52–55). Further work investigating neuronal insulin resistance has identified downregulation of the insulin receptor β-subunit, upregulation of glycogen synthase kinase 3β, and impairment of Akt activation (51). Transcript analysis suggested that while T1 and T2DM related DPN share the JAK-STAT signaling pathway, lipid and cholesterol metabolism could be more relevant in T1DM mice, and MAPKinase NF-kB signaling more important in T2DM (56). The implications of this shift on potential treatments for DPN are unclear. In the type 1 BB/Wor-rat model, the polyol pathway was activated, impairing Na+/K+-ATPase function (57). However, studies by the same investigators using type 2 BBZDR/Wor-rats showed polyol pathway activation but without Na+/K+-ATPase impairment (48). C-peptide is the portion of the insulin molecule that connects the α and β chains, is cleaved from mature insulin, and is absent from insulin deficient T1DM. However, it may also be biologically active and has been advocated as exogenous therapy for DPN. Administration of C-peptide in type 1 BB/Wor-rats restored Na+/K+-ATPase activity (58). Further, C-peptide administration in type 1 BB/Wor-rat improves features of DPN without correcting oxidative stress (49). Since C-peptide levels in T2 DM may be normal or high, this approach might be less helpful in T2 DM.

### Metabolic impairment

**Figure 1:**
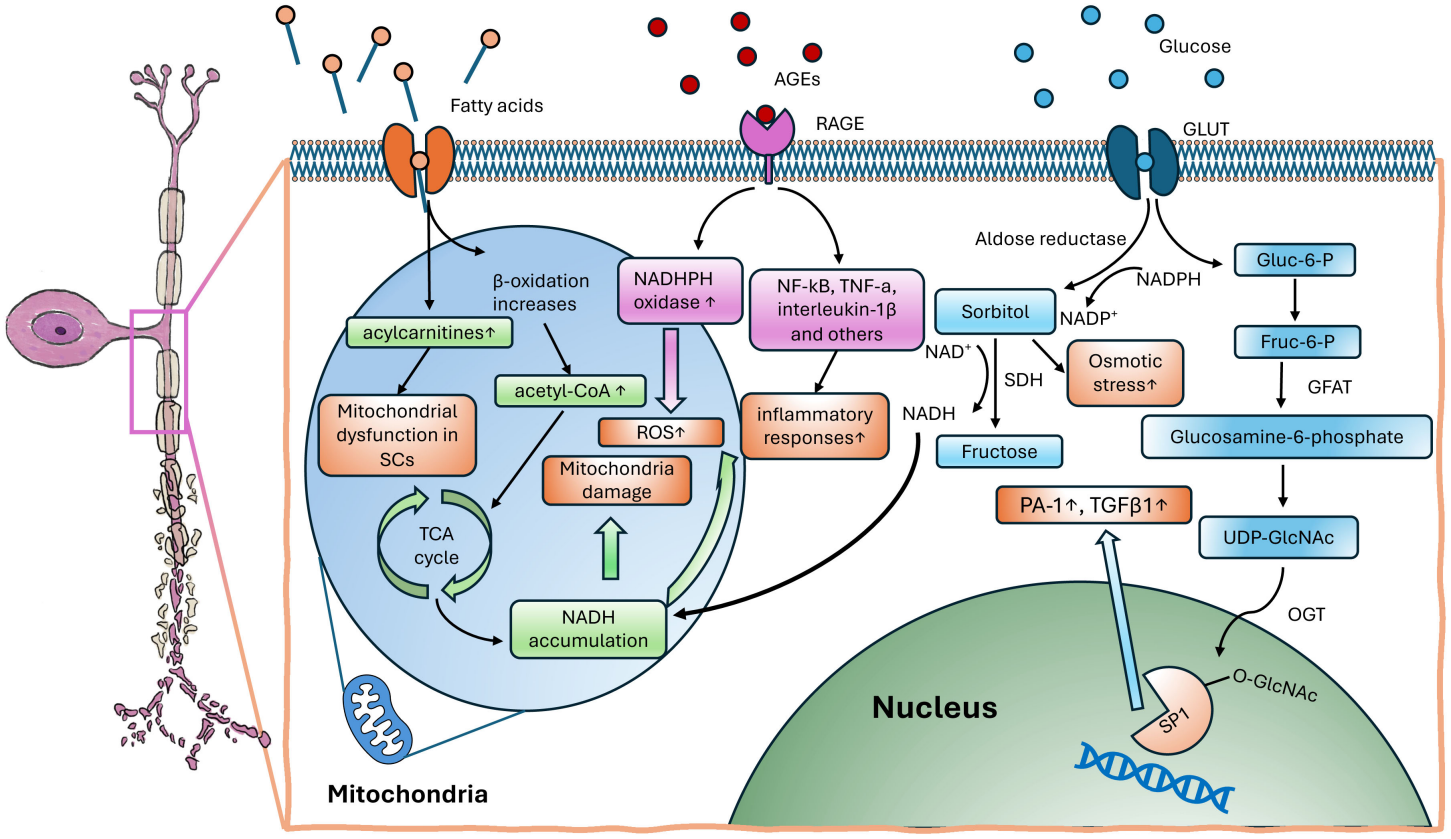
Metabolic mechanisms contributing to diabetic polyneuropathy (DPN). Metabolic disorders in diabetes, including hyperglycemia, hyperlipidemia, and advanced glycation, contribute to neuronal and glial dysfunction through multiple pathways. Hyperglycemia activates the hexosamine pathway, in which glucose is converted through glucose-6-phosphate (Gluc-6-P), fructose-6-phosphate (Fruc-6-P), and glucosamine-6-phosphate by glutamine:fructose-6-phosphate amidotransferase (GFAT), leading to the production of uridine diphosphate N-acetylglucosamine (UDP-GlcNAc). UDP-GlcNAc is a substrate for O-GlcNAc transferase (OGT), promoting glycosylation and activation of specificity protein 1 (Sp1), which increases the expression of plasminogen activator inhibitor-1 (PAI-1) and transforming growth factor-β1 (TGF-β1). These changes may contribute to microvascular dysfunction, apoptosis, and axonopathy through increased reactive oxygen species (ROS) generation. Excess intracellular glucose is also metabolized through the polyol pathway, where aldose reductase converts glucose to sorbitol, promoting osmotic stress, followed by conversion of sorbitol to fructose by sorbitol dehydrogenase (SDH). This pathway also consumes nicotinamide adenine dinucleotide phosphate (NADPH), generates nicotinamide adenine dinucleotide (NADH), and causes oxidative stress. Chronic hyperglycemia additionally leads to the formation of advanced glycation end-products (AGEs), which bind to the receptor for advanced glycation end-products (RAGE), activating nuclear factor kappa B (NF-κB), tumor necrosis factor-α (TNF-α), interleukins, and NADPH oxidase, thereby enhancing inflammatory responses and ROS production. Hyperlipidemia contributes to DPN through increased fatty acid β-oxidation. Excessive β-oxidation increases acetyl-coenzyme A (acetyl-CoA) levels, disrupting tricarboxylic acid (TCA) cycle activity and oxidative phosphorylation. The resulting accumulation of NADH promotes mitochondrial dysfunction, increased ROS production, and mitochondrial damage. As an alternative pathway, excessive fatty acids are converted into acylcarnitine. Acylcarnitine accumulation may further induce maladaptive stress responses in Schwann cells (SCs), contributing to demyelination and axonal degeneration. Together, these metabolic abnormalities converge on oxidative stress, inflammation, mitochondrial dysfunction, and energy failure, ultimately promoting peripheral nerve injury in DPN.

Metabolic disorders associated with DM include hyperglycemia, hyperlipidemia, altered insulin production or signaling (resistance), ketosis, glycation and other changes (7) (**Figure 1**). Hyperglycemia can alter a number of intracellular pathways. For example, the hexosamine pathway transfers glucosamine-6-phosphate to uridine diphosphate-n-acetylglucosamine (UDP-GlcNAc), to eventually upregulate specificity protein 1 (Sp1) (59). Sp1 activation, in turn, can result in fibrinogen activator inhibitor-1 and transforming growth factor-β overexpression. Downstream potential impacts may include microvascular thrombosis, apoptosis and axonopathy through exposure to ROS (reactive oxygen species) (60, 61). Excess glucose can also be converted into sorbitol through the polyol pathway, altering osmotic balance (perhaps acutely only) and generating ROS (40, 62). However, ROS involvement is implicated in a large variety of neurological disorders from stroke to neurodegeneration. Given this, the advocacy of therapeutic options that include routine inhibition of oxidative stress may be harmful (63). Tissue function depends on a careful redox balance (61).

Chronic exposure to hyperglycemia leads to protein and lipid formation of advanced glycation end-products (AGEs) (64, 65), that bind to advanced glycation end-product receptors (RAGEs). RAGE binding can activate pro-inflammatory markers, potentially leading to microvascular injury and glial cell damage (66). RAGE deletion attenuates experimental DPN in chronic T1DM (STZ) mice (67). However, it is also noted that RAGE activation promotes neurite outgrowth through NF-κB, JAK-STAT or ERK pathways (68), rendering controversy about how we should tune AGE pathways to protect axons.

Hyperlipidemia may contribute to DPN given its prevalence in the prediabetic metabolic syndrome. This syndrome is associated with small fiber polyneuropathy in the absence of a frank diagnosis of DPN but with evidence of insulin resistance (69, 70). Severe triglyceridemia alone, requiring far higher lipid levels, without hyperglycemia has also been linked to a form of small fiber polyneuropathy but it is an uncommon diagnosis (63). These observations suggest that more than hyperlipidemia alone is required to develop neuropathy. During hyperlipidemia, β-oxidation may convert lipids into acylcarnitine and away from myelin synthesis, accumulation of which may generate a maladaptive integrated stress response and mitochondrial dysfunction in SCs. This pathway may also target sensory neurons, eventually leading to nerve degeneration (71). In an oversaturated system, acetyl-CoA from β-oxidation can inhibit the citric acid cycle impairing oxidative phosphorylation leading to reduced ATP production, enhanced ROS production, compromised mitochondria function, and energy depletion (72). Meanwhile, free excessive fatty acids also produce ROS and recruit macrophages, causing focal and general inflammation that may exacerbate DPN (73).

### Insulin

Insulin receptors (IRs) have been localized to sensory neuron perikarya, nerve trunk axons, dermal/epidermal skin axons, and SCs (74–76). Direct insulin signaling attenuates DPN by providing trophic support through phosphoinositide 3-kinase (PI3K)–protein kinase B (Akt) signaling pathways (77). After ligand binding, IRs undergo autophosphorylation, that next activate IRS-1 and IRS-2. IRS proteins and PI3K actuate the generation of phosphatidylinositol (3–5)-trisphosphate (PIP3). PDK1 (3-phosphoinositide-dependent protein kinase-1) is then recruited, phosphorylating and activating Akt (78). Akt also supports SC differentiation, myelination and axon growth (79, 80). Insulin supports myelination in part through its rescue of lipoprotein lipase (LPL) but its impact in T2DM is attenuated due to insulin resistance (81). Sensory neurons exposed to prolonged incubation with insulin or high dose insulin develop resistance to its trophic actions (51). Its role is discussed in more detail below.

### The role of microvascular impairment

As described in diabetic retinopathy and nephropathy, DPN has been labeled as a microvascular complication, a designation that may detract from the unique targeting of neurons and SCs (82). Microvascular pathology is well documented in chronic human disease but its role as a pathogenic trigger in DPN has been controversial (42, 83). Nonetheless, it is well established that endothelial damage in vessels may promote vasoconstriction (84). Declines in nerve or ganglion blood flow reduction accompanied by declines in oxygen tension may contribute toward secondary nerve dysfunction and degeneration (85–87). In longer duration DM in humans, microvessels of the sural nerve develop thickened capillary membranes, degenerated pericytes and hyperplastic endothelium (88, 89). Intimal area is greater in the epineural arteriolar wall in diabetic patients compared to nondiabetic control nerves (90). Despite these findings, several preclinical studies and direct sural nerve blood flow recordings in humans with DPN have not identified declines in nerve blood flow. For example, serial analysis of sural nerve blood flow using laser Doppler flowmetry did not observe a decline over a period of one year despite progressive features of DPN (91). An additional hypothesis has suggested that there may be variable capillary perfusion without a global decline in overall blood flow, that causes capillary transit time heterogeneity (CTH) (88). Early studies of vasodilation did not improve experimental DPN in rats (92). Finally, contrary findings of increased epineural blood flow and blood oxygen saturation in the sural nerve have been observed in patients with painful DPN, a finding that is difficult to reconcile (93, 94).

### Mitochondrial dysfunction

Chronic hyperglycemia and hyperlipidemia may promote excessive ROS production through aberrant metabolic pathways, leading to oxidative stress and cellular dysfunction, discussed above (**Figure 1**). In turn, dysregulated ROS generation can impose substantial stress on mitochondria, the endoplasmic reticulum (ER), and the nucleus in T1DM (95). Thus, strategies to quench pathological ROS might offer protection of neurons and axons. Moreover, the drug metformin, a first-line therapy for T2DM, can impair folic acid and vitamin B12 absorption, vitamins that limit ROS damage (96). Lipid peroxidation-induced ferroptosis, a form of programmed cell demise, was improved in DPN mice by rosiglitazone, a commonly used anti-glycemic agent (97). ROS damage may particularly target mtDNA indicating a vulnerability of mitochondria to oxidative stress (98). Normally, the ROS mediator H_2_O_2_ is precisely maintained in redox homeostasis through catalase activity. Due to its relatively low affinity for H_2_O_2_, catalase primarily functions under conditions of elevated H_2_O_2_ concentrations. However, catalase activity is reduced in diabetes (99). The polyol pathway may then act to consume NADPH during hyperglycemia, depletion impairing the capacity of neurons to attenuate oxidative stress (100). Autophagy is observed in SCs with excessive ROS (101). Furthermore, a specific type of autophagy, mitophagy, is responsible for removing dysfunctional or damaged mitochondria. During this process mitochondria fission may contribute by facilitating autophagosome encapsulation (102).

Along these lines, the PTEN-induced kinase 1 (PINK1)–Parkin pathway, best known for its role in Parkinson’s disease, participates in mitophagy. Upon mitochondrial depolarization, PINK1 accumulates on the outer mitochondrial membrane, where it recruits Parkin to damaged mitochondria in neuronal cells. Parkin subsequently ubiquitinates outer mitochondrial membrane proteins such as VDAC1, leading to the recruitment of p62/SQSTM1 and the initiation of mitophagy (103). Diabetic hyperglycemia can increase the expression of Drp1 (dynamin-related protein1) (104–106) in DRG neurons, promoting mitochondrial fission (107). Drp1 may also interact with BAX, a Bcl-2 family apoptotic-related protein, promoting mitochondrial outer membrane permeabilization (MOMP) and further activating caspases and apoptosis pathways (108). Drp1-mediated beta cell apoptosis has been observed in diabetic beta cells (106). Mitochondrial inner membrane depolarization has been reported in neurons of streptozotocin (STZ)-induced diabetic mice but direct diabetic neuronal apoptosis, described in this work, has been a debated outcome (109). Insulin impacts mitochondrial function, and is reported to attenuate mitochondrial membrane depolarization through PI3K (110). In rats after 22 weeks of diabetes, DRG homogenates and purified mitochondria exhibited abnormalities in mitochondrial bioenergetics, including impaired oxidative phosphorylation, uncoupled respiration, and reduced Complex IV electron transport activity. All these deficits were ultimately prevented by insulin (111). Recent work also describes a novel impairment of perineuronal satellite glial cell (SGC) to DRG sensory neuron mitochondria transfer in diabetic mice. SGC-derived myosin 10 was noted to promote tunneling nanotube formation that is required to transfer mitochondria (112). Overall, these deficits are thought to impair neuronal energy supply with excess oxidative stress, resulting in DRG neuronal injury.

### Mechanisms of neuropathic pain in DPN

A study of European, Asian, and African populations suggested that 34% of diabetic patients had painful neuropathic symptoms, while 24% were diagnosed with painful diabetic neuropathy (113). The prevalence of pain was 26% in persons without neuropathy, rising to 60% in advanced neuropathy. The risk of painful neuropathic symptoms in T2DM was twice that in T1DM but the reasons for this finding are not clear (113). It is also unexplained why pain is prominent in some DPN persons but not others. Genome-wide association studies (GWAS) identified a 11-15% genetic risk for painful DPN and 6% for diabetic foot ulcers (114–116). Three specific genetic targets of neuropathic pain from studies of a Scottish population included single nucleotide polymorphisms (SNPs) associated with GFRA2 (Glial cell line-Derived Neurotrophic Factor Family Receptor Alpha 2;odds ratio=0.67), TLR12P (Toll Like Receptor 12;odds ratio=1.66), and HMGB1P46 (High Mobility Group Box 1 Pseudogene 46;odds ratio=1.67) (114, 115). GFRA2 is expressed on cutaneous nociceptors whereas TLR12P is an inflammatory marker and HMGB1P46 is a noncoding gene (117). Further GWAS analysis identified relationships of SNPs near NOS1AP (β=−0.77), and KCNT2 (β=0.51) with painful diabetic neuropathy (118). NOS1AP and KCNT2 encode nitric oxide synthase 1 adaptor protein and sodium activated potassium channel respectively (119, 120).

Epigenetic associations have also been identified. For example, genome-wide assessment of DNA methylation coupled with transcript expression in T2DM human sural nerves identified a series of altered methylomes. These were differentially altered depending on glycemic control (HbA1C) and in some instances, suggested less control of specific gene expression, findings that could contribute to neuropathic damage (121). DNA methylation of GCH1 (GTP cyclohydrolase 1), MYT1L (Myelin Transcription Factor 1-Like), and MED16 (Mediator of RNA polymerase II transcription subunit 16) differentiated painful and painless type 2 diabetic neuropathy, indicating specific molecular loci that might have a role in modulating pain (122).

Higher levels of endothelial cell activation and systemic inflammation markers including soluble Intercellular Adhesion Molecule 1 (slCAM-1) and C-reactive protein (CRP) have been reported in patients with painful DPN (123). Other associated changes have been rises in tumor necrosis factor-alpha (TNF-alpha) and reduced vitamin D and Glo-1(glyoxalase 1), an enzyme that breaks down methylglyoxal, a potential mediator of axon damage (124) (125**)**.

Cutaneous nerve fiber regeneration is positively correlated with pain in T2DM, although the extent of regrowth failed to restore innervation (126). In keeping with this idea, the GAP43/PGP9.5 ratio, a regeneration index, was higher in painful diabetic than painless neuropathy. Finally, painful DPN was associated with a higher ratio of swollen axons, a change that may indicate early evidence of degeneration (127). This interesting evidence for an association between pain and nerve regeneration differs from conventional views that neuropathic pain is associated with nerve fiber loss (128). The significance of these changes and how they trigger pain remains uncertain. Axon loss alone might be expected to lessen pain generated by ectopic discharges from damaged axons. However axons (or their perikarya) undergoing an influx of degeneration and regenerative signals may be triggered to generate inappropriate pain discharges. While not discussed here, these pathways likely do involve participation of cytokines linked to other forms of pain. Taken together, these distinctions may provide new insights and perhaps differing therapeutic avenues for pain (129) management. Overall, dysfunctional predegenerative but preserved axons or those with truncated or frustrated regeneration may be contributors to the pain syndrome.

## Novel molecular targets in sensory neurons

New treatment for DPN, focused on the rescue of sensory neurodegeneration requires attention to two goals: i) restoration/regrowth of neurons and ii) neuroprotection, preventing axon and potential neuron loss. We believe these molecular impacts overlap, albeit not completely. For example, insulin, discussed below, enhances growth and prevents loss of epidermal axons but these dual roles only emerge in specific testing paradigms. Regenerative growth can be probed *in vitro* by assaying neurite outgrowth, or in early regrowth after injury in a model *in vivo*. In the context of growth cones that may actively advance or retract, this linkage becomes more obvious-regeneration is linked to arresting retraction simultaneously with advancing growth cone filopodia and lamellipodia. Here, we examine several genes/proteins that may emerge as translational targets for DPN. Most are discussed in the context of growth or regenerative promotion but those linked more closely with neuroprotection are considered separately. A compilation of new targets is summarized in **Table 1**.

**Table 1:** Diabetic polyneuropathy: translational landscape.

Agents	Impacts	Drawbacks	Status	References
Neuron growth promotion				
Growth Factors: NGF, BDNF, NT3, HGF	Trophic support of sensory axons	Pain from rhNGF; HGF no impact on IENF	No reported human benefits from NGF, BDNF, NT3; HGF improved pain; extensive preclinical work (not listed)	(130–134)
Intracellular growth pathways: PTEN, Rb1,APC, Mad1 inhibition	PTEN, Rb1 and APC inhibit growth signaling; Mad1 inhibits Myc growth signaling	Requires siRNAs for Rb1, APC, Mad1; siRNAs and small molecule inhibitors available for PTEN	Preclinical work only; all show impact in regeneration models; PTEN KD, inhibition tested in DPN models;	(135–139)
Low dose insulin: topical or intranasal	Ligates neuronal insulin receptors and promotes growth	Insulin resistance of neurons	Preclinical work only: Local, intranasal, intrathecal insulin impacts DPN models; Insulin additive to PTEN KD;	(67, 76)
GLP1- agonists	Ligates neuronal receptors and promotes growth	Systemic side effects; only approved for T2 DM	Preclinical data; Mixed small trial results confined to T2DM persons	(50, 140) (10) (141) (142) (143) (144)
Local growth cone facilitation				
Muscarinic antagonists	Inhibits Ach signal suppression of axon growth	Systemic side effects but avoided by topical treatment	Phase II human trial in DPN increased IENF and some QOL measures	(145–147)
Rac1 activation	Enhances growth cone advancement	Requires local action	Preclinical work in regeneration only	(148)
RhoA inhibition	Inhibits growth cone retraction	Systemic impacts uncertain	Preclinical work in regeneration only	(149)
Pcdhγ inhibition	Pcdhγ is a repulsive intracellular adhesion molecule. Inhibition (siRNA) facilitates axon growth and skin reinnervation	Local therapy only; Long term impact uncertain	Preclinical work in regeneration only	(150)
Local insulin-see above	Activates insulin receptors on sensory axon terminals	May develop Insulin resistance	Preclinical work in DPN models only	(76, 151)
Axon protection				
SARM1 inhibitors	Attenuates the axon degenerative cascade	Other systemic impacts of SARM1 unclear	Phase 1–2 trials in other conditions	(152–155)
Caspase-6 inhibitor	Inhibits later stage (than Sarm1) axon degeneration	Systemic impact Unexplored; siRNA and genetic models	Preclinical work in injury model only	(156)
Metabolic therapeutics				
Aldose reductase inhibitors (ARIs)	Reduces sorbitol; Restores Na/K ATPase-myo-inositol	Overall Cochrane review negative impact; renal, hepatic, hypersensitivity side effects in 3 agents	Most trials discontinued (see Cochrane review of 32 trials)	(9, 157)
RAGE inhibition	Inhibits AGE activation of its receptors (RAGE)	Pathway has mixed impacts	Preclinical work in genetic models	(67)

Other pharmaceuticals*: rosuvastatin, CGRP antagonism (pain), thioctic (alpha-lipoic; several trials) acid, lidorestat (ARI),ranirestat (ARI), C-peptide, topiramate, dapagliflozin;.

For Pain: topical capsaicin, novel opioids, botulinum toxin, cilostazol, duloxetine/pregabalin/tramadol,carisbamate, other proprietary trials

Not reviewed*: dietary supplements, rehabilitative strategies, stimulation techniques, vitamin therapy, laser therapy, clothing, acupuncture, thermal therapy, vibration therapy, ultrasound* selected from 151 studies listed on ClinicalTrials.gov (June 24, 2026).

AGEs, advanced glycosylation endproducts; APC, adenomatous polyposis coli; BDNF, brain-derived neurotrophic factor; DPN diabetic polyneuropathy; HGF, hepatic growth factor; IENF, intra-epidermal nerve fibers [density]); KD, knockdown (siRNA), NGF, nerve growth factor; NT3, neurotrophin 3; PTEN, phosphatase and tensin homolog deleted on chromosome 10; RAGE, receptor for AGEs; Rb1, retinoblastoma 1.

### Growth-promoting agents

*Phosphatase and Tensin Homolog deleted on chromosome 10 (PTEN)* is a largely cytoplasmic tumor suppressor protein that regulates cell growth, division and survival by inhibiting the PI3K/AKT pathway (158). PTEN is a phosphatase enzyme that acts through dephosphorylation of PIP3 into PIP2, thus preventing the activation of Akt to pAkt. This ‘brake’ on growth is essential to prevent uncontrolled growth and cell proliferation. PTEN is also found localized in the nucleus, maintaining chromosomal stability and regulating the cell cycle (159). Dysregulation or loss of PTEN leads to uncontrolled cell proliferation, contributing to a number of solid malignancies (160). In both T1 and 2DM mouse models (STZ, db/db), PTEN expression was elevated in the dorsal root ganglia (DRG) sensory neurons compared to those of littermate controls. In contrast, PTEN expression is downregulated after injury in nondiabetic neurons, indicating development of a regenerative cell phenotype. In DM, elevated PTEN levels may act to suppress regenerative regrowth (161), but also to limit the repair of deficits in DPN (162). PTEN inhibition supports the growth properties of both nondiabetic and diabetic neurons as analyzed in dissociated cultures *in vitro* (135, 161). *In vivo*, PTEN knockdown using siRNA improved recovery of compound motor action potentials (CMAPs) and sensory nerve action potentials (SNAPs) in nondiabetics and diabetic mice following nerve injury. There were more regenerating myelinated axons distal to the injury site, better recovery of mechanosensation and improved reinnervation of the hindpaw epidermis (135, 161).

**Figure 2:**
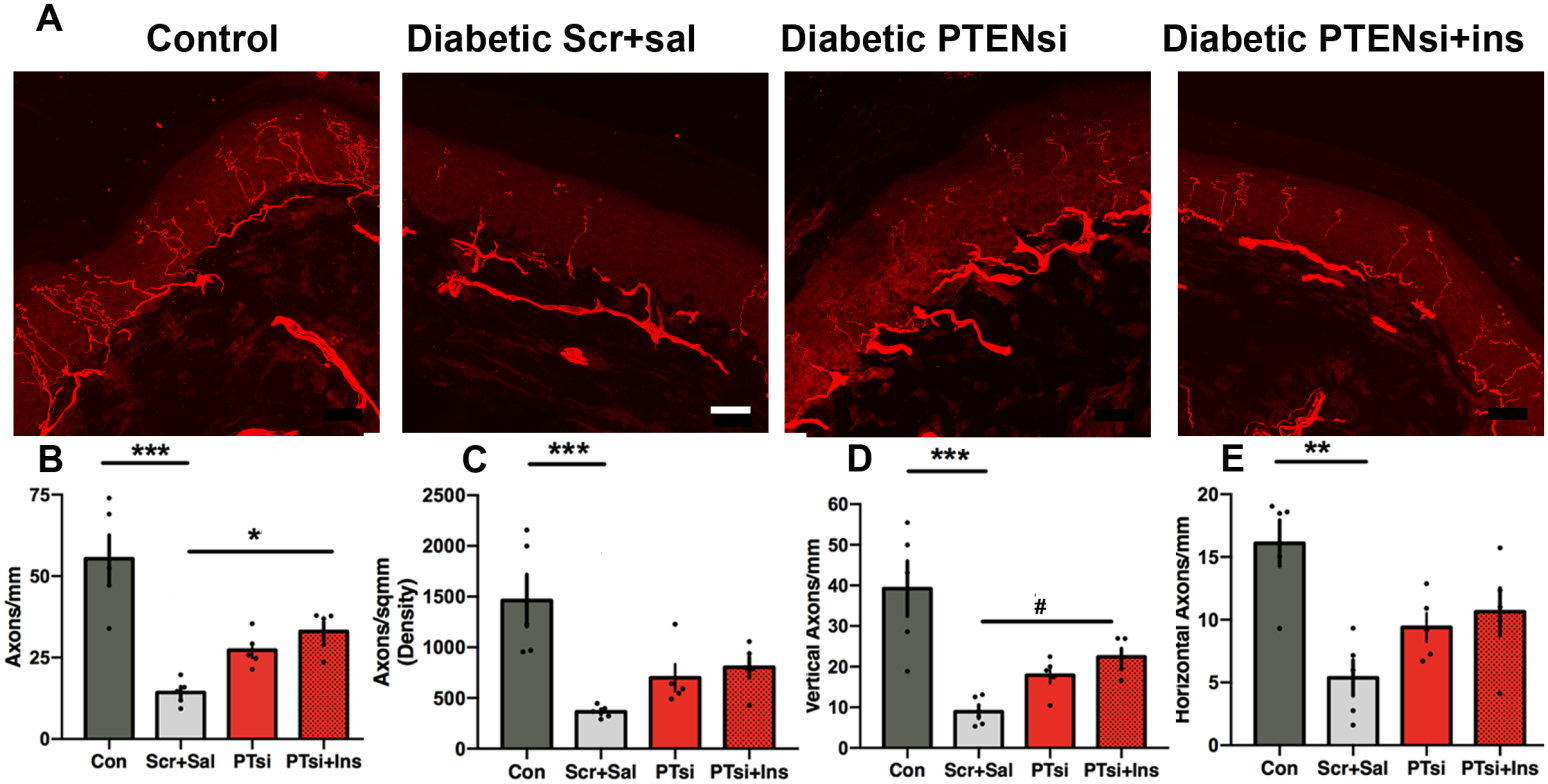
PTEN KD with insulin (Ins) repairs morphological abnormalities in chronic experimental diabetes. **(A-E)**- Skin reinnervation of the epidermis is depicted with PTEN KD combined with insulin. These are examples of epidermal innervation in panel **(A)** (Bar = 25 μm) and **(B-E)** provide quantitation of axon innervation of the skin expressed as axons per length of skin **(B)**, axon density **(C)**, vertically oriented (>45°from plane of skin) per length of skin **(D)**, and horizontally oriented per length of skin **(E)**. Diabetes was associated with axon loss. PTEN si alone and PTEN si + insulin repaired axons per length, axon density, and vertical axons per length. (In panel B, *P* < 0.0001 by one-way ANOVA with Tukey *post hoc* test, ***Nondia vs. Dia Scr + sal, *Dia Scr + sal vs. Dia PTEN si + Ins. In panel C, *P* = 0.0011 by one-way ANOVA with Tukey *post hoc* test, ***P = 0.0007 Nondia vs. Dia Scr + sal. In panel D, *P* = 0.0003 by one-way ANOVA with Tukey *post hoc* test, ***Nondia vs. Dia Scr + sal; pairing ipsilateral and contralateral #*P* = 0.014 Dia Scr + sal vs. Dia PTEN + Ins. In panel E, *P* = 0.0022 by one-way ANOVA with Tukey *post hoc* test, **Nondia vs. Dia Scr + sal (*n* = 5 all axon comparisons all groups). (Reproduced with permission from the American Diabetes Association, Diabetes, through the Copyright Clearance Center). The citation is (164).

The facilitation of axon outgrowth from PTEN knockdown or inhibition may be independent of canonical mTOR signaling and was unaltered by rapamycin, an mTOR inhibitor (135). Further mediators of PTEN inhibition include pAkt suppression of GSK3β within the perikarya, leading to stabilization and prolonged Smad1 signaling, promoting axon growth. Smad1 alone, however, may be insufficient to enhance regeneration (163, 164) but collaboration of phosphorylated Smad1 with histone-acetylating enzymes facilitates the activity of regeneration-associated genes (RAGs) (165). PTEN inhibition and local insulin appeared to show additive impacts on the plasticity of sensory neurons, further improving axon regeneration and improvement of DPN in mice. These studies were carried out in chronic T1DM STZ mice. Benefits included recovery of motor conduction velocities, thermal and mechanical sensation and epidermal innervation in DPN mice (136) (**Figure 2**). The most apparent caveat in mobilizing this pathway may be its theoretical facilitation of oncogenesis. However, oncogenesis is a multi-hit process and PTEN knockdown that is targeted and restricted in duration may offer benefits in post mitotic neurons (166). Long term PTEN deletion is also associated with inappropriate axon sprouting in the absence of injury and changes in cortical neuronal properties (120, 129, 167–170).

*Retinoblastoma (Rb1) protein* is also a tumor suppressor molecule, and its canonical role is to regulate the cell cycle by binding and inhibiting E2F transcription factors during the G1 phase, thus arresting cell division. Mutations of Rb1 are associated with retinoblastoma, osteosarcoma, melanoma and other tumors (171). Nonetheless, physiological levels of Rb1 are widely expressed in DRG sensory neurons including nuclei and axons. Rb1 knockdown *in vitro* is associated with increased neurite outgrowth and improved regeneration outcomes *in vivo*. These impacts were also evident when applying Rb1 knockdown in a delayed fashion, once initial sprouting was underway. Thus, the approach was impactful after the regenerative cascade following nerve injury had already been initiated (137). There were additive impacts when combined with local insulin, as described above with PTEN knockdown (172). Downstream mediators from Rb1 knockdown include PPAR-γ, a mediator that may account for some, but not all of the regenerative impacts of Rb1 knockdown (137).

Thiazolidinediones (TZDs) are a class of PPAR-γ agonists, which have been used clinically to treat T2DM. TZDs bind to PPAR-γ receptors and regulate gene expression to reduce insulin resistance in adipose tissue, muscle and liver (172). Thus, PPAR-γ agonists may not only offer benefits for DPN through its impact on glycemic control but also in improving intrinsic growth capacity of neurons (137). TZDs are further thought to be neuroprotective by enhancing mitochondrial function, increasing amyloid clearance and reducing neuroinflammation (173–176). Preclinical studies of Rb1 knockdown in diabetic models is awaited, but like PTEN, theoretical oncogenic impacts exist, but can be mitigated. Risks might arise from prolonged usage and nonselective targeting (177, 178**).** Given the combined findings that Rb1 knockdown promotes axon regeneration, and PPAR-γ agonists act as insulin sensitizers and neuroprotectors, Rb1 inhibition together with PPAR-γ agonists may offer translational opportunities in treating DPN. However, impacts have not yet been reported in DM models.

*Adenomatous polyposis coli (APC)* is a further tumor suppressor molecule whose manipulation may foster regenerative regrowth in DPN and other conditions. APC suppresses the canonical Wnt/β-catenin nuclear signaling pathway, participating in a destruction complex preventing β-catenin translation to the nucleus. β-catenin is a critical transcription factor involved in cell proliferation, differentiation and apoptosis (179). In injured peripheral sensory neurons, APC is paradoxically upregulated, a change that attenuates regrowth, whereas its knockdown following nerve injury improved regeneration. APC siRNA administration was associated with increased neurite outgrowth *in vitro* and greater regrowth and maturation of axons *in vivo* after injury (180). This pathway also awaits preclinical work in DM models.

### Peptide hormones

*Local insulin* acts beyond its traditional role in glycemic control, operating as a neurotrophic factor that promotes axon growth (181, 182**).** Although neurons do not take up glucose in an insulin-dependent manner, many neuronal populations express insulin receptors (183). In the PNS, insulin receptors are expressed on DRG neuron perikarya as well as in the peripheral nerves and their terminals (184, 185). Insulin supplementation in DRG sensory neuron cell culture led to increased neurite outgrowth that was additive with the actions of nerve growth factor (80). Further, insulin signals through the PI3K-Akt pathway, which is directly related to axonal growth and neuron survival as discussed above (186). Following sciatic nerve crush, systemic administration of insulin through intraperitoneal injections in rats accelerated myelinated fiber regeneration and recovery of nondiabetic mouse hindpaw function (187). Alternatively, rats with euglycemia that received subdiabetic doses of STZ with partial declines in insulin levels developed mechanical hyperalgesia resembling findings of rats with frank hyperglycemia and DM. These changes were rescued with supplemental low doses of systemic insulin (188).

Systemic insulin (0.4 IU daily by pump) administered over one month improved hyperglycemia but only partly reversed features of DPN in T1DM (STZ) mice that had been diabetic for two months. In contrast, db/db T2DM mice undergoing similar treatment had no improvement in hyperglycemia or neuropathy despite larger insulin doses (up 1.2 IU daily). Low systemic doses of insulin (0.1 IU daily) did not improve glycemia or neuropathy in either T1 or T2 DM mice (50). In contrast, low dose intranasal insulin (0.1 IU daily) improved features of T1 STZ induced DM in mice with important differences in the responses of male and female mice, a biological variable not often studied in these preclinical models (67). RAGE null mice with T1 STZ DPN had attenuated neuropathy with only limited added impacts of intranasal insulin, indicating overlap in these pathways (50). Intrathecal delivery of insulin daily for 4 weeks restored motor and sensory nerve conduction in a T1DM STZ rat model and rescued loss of their epidermal axons (75, 189). In addition, local intraplantar injection of low dose insulin reversed the loss of epidermal innervation in types 1 and 2 DPN mice (76). DPN is associated with elevated reactive oxygen species (ROS), as discussed above, and impaired mitochondrial bioenergetics that may contribute to neuron degeneration (190). Insulin signaling is critical for its support of mitochondrial function. Insulin treatment in DPN models prevents excessive depolarization of the mitochondrial inner membrane and corrects mitochondrial respiratory chain dysfunction (110, 111).

*Glucagon-like peptide-1 (GLP-1)* is an incretin hormone secreted from enteroendocrine L-cells in response to digestion of food. GLP-1 stimulates insulin production, inhibits glucagon release and slows gastric emptying. It is a key player in regulating glycemia and promoting satiety (191). However, its potential as a therapeutic is limited due to its short biological half-life, primarily degraded by dipeptidyl peptidase IV, an enzyme widely distributed on the surface of a number of cell types but also as a soluble form in body fluids (192). To circumvent this, GLP-1 receptor agonists (GLP-1 RA) have been designed and widely adopted to treat T2DM and obesity. Examples include semaglutide, tirzepatide, exenatide and others. GLP-1 receptors are also expressed on sensory neurons, SCs, sympathetic neurons, vagal afferents and the enteric nervous system (193). In STZ-induced diabetic rats, intraperitoneal injections of synthetic exendin-4 (exenatide) over 24 weeks improved current perception sensation, a surrogate behavioral test, improved epidermal innervation and reduced the number of cleaved caspase 3-stained SCs (192). Cleaved caspase-3, however, has been identified in sensory neurons of diabetic rats without associated cell loss, indicating a form of cellular stress not necessarily equivalent to cell apoptosis (194, 195). Detailed preclinical work in 2 month T1DM (STZ) and T2DM (dbdb) mice confirmed the presence of GLP-1 receptors on sensory neurons and compared the impact of exendin-4 and insulin on behavior and electrophysiology after one month of treatment in both models (50). In this paradigm, the GLP-1 agonist underwent back to back comparisons with low doses of insulin or alternative higher insulin doses designed to reverse hyperglycemia. The high continuous doses of insulin by pump normalized glycated hemoglobin but exendin-4 had no impact on glycemia or glycated hemoglobin. Despite the absence of an impact on glycemia, exendin-4 had greater impacts on experimental DPN than either form of insulin treatment in T1DM mice. Specifically, there were improvements in motor conduction velocity, mechanical and thermal sensation with exendin-4. In contrast, following high doses of insulin over one month, there were no improvements in conduction velocities, with more mild improvements in thermal and mechanical sensation. Insulin had no impact on T2DM mice, whereas exendin-4 improved motor conduction velocity but not other indices of DPN. However, high continuous insulin treatment failed to improve glycated hemoglobin in these mice, indicating insulin resistance.

Clinical studies have emerged more recently. This work has focused on T2DM persons who, unlike persons with T1DM, are candidates for GLP-1 agonist therapy. Findings from a small clinical trial of T2DM subjects (n=24) using a GLP-1 RA (dulaglutide, semaglutide or exenatide) demonstrated improvement in total neuropathy score (TNS), nerve conduction measurements and axonal excitability recordings over 3 months (10). This followed an earlier study showing improvement in nerve excitability in 10 persons with T2DM prospectively studied over 3 months with exenatide therapy (142). Finally, a parallel study by the same group in 22 T2DM patients using dulaglutide or semaglutide for 1–3 months, showed improvement of an ultrasound measure of tibial nerve size, the sural SNAP and symptoms (141). However, an 18 month open label proof of concept trial of exenatide in 22 treated T2DM patients was negative (143). A further trial of 38 T2DM persons treated with exenatide and pioglitazone or basal bolus insulin over one year improved corneal innervation but not DPN symptoms or sudomotor (sweat) autonomic function (144). Overall these are mixed results. Investigators and authors have hypothesized that possible benefits from GLP-1 agonists may be related to attenuation of pro-inflammatory cytokines, enhancement of mitochondrial function and activation of PI3K/Akt and MAPK/ERK pathways (196–199**).** Due to the possible neuroprotective effects offered by GLP-1 RAs, they are also under investigation for neurodegenerative diseases such as Alzheimer’s and Parkinson’s disease (200).

### Locally acting interventions for DPN

There is likely a range of additional growth-promoting agents that may offer opportunities to reverse the axon deficits of DPN. For example, axonal guidance cues, mainly operating at the axonal growth cone, regulate attraction or repulsion signals to determine the pathway of axons during regrowth (201). These signals act on intrinsic molecules within the growth cones but may be offered by target cells, such as keratinocytes. Growth cones express Rho GTPases that inhibit growth specifically, such as RhoA or alternatively facilitate outgrowth such as Rac1 or CDC42. While not yet studied in experimental DPN, their manipulation supports peripheral neuron regrowth as tested both *in vitro* and *in vivo*. Pharmacological inhibition of RhoA is associated with greater neurite outgrowth and regeneration after nerve injury (149). In contrast, activation of Rac1 enhances *in vitro* neurite outgrowth. Its local application promotes regrowth of sensory axons to support enhanced reinnervation of the epidermis (148).

*Protocadherin-gammas (Pcdh-γ)* are a group of homophilic cell adhesion proteins, primarily expressed in the mammalian nervous system. In the central nervous system, Pcdh-γ has been implicated in the establishment of neurocircuitry by regulating neuronal survival, dendrite arborization as well as synaptic development (202–204). In the PNS, *in vitro* knockdown of Pcdh-γ in sensory neurons was associated with increased neurite outgrowth and branching. Following sciatic nerve crush in mice, knockdown of Pcdh-γ through intraplantar injection of siRNA led to increased intraepidermal reinnervation (150). Given these findings, Pcdh-γ acts as an apparent brake on regrowth of axons into the skin, perhaps mediated by axon-keratinocyte repulsion. Removal of this novel local brake may be an important strategy to improve reinnervation of the skin. This has not been tested in DM models.

*Antagonism of muscarinic type 1 receptor (M_1_R)* offers an alternative approach to foster skin reinnervation through its local actions on cutaneous axons. Muscarinic acetylcholine receptors (mAChRs) are members of the superfamily of G-protein coupled receptors, consisting of 5 molecular subtypes: M_1-5_R. M_1_R couples with G_q_ to activate the inositol triphosphate (IP_3_) pathway, leading to increased intracellular calcium (205). In mouse DRG neurons, antagonism of M_1_R through pirenzepine (a selective M_1_R antagonist) or muscarinic toxin 7 (MT7, a specific M_1_R antagonist) led to enhanced neurite outgrowth *in vitro*. Alternatively, an M_1_R agonist, muscarine or overexpression of M_1_R caused inhibition of neurite growth, indicating a role of M_1_R in tonic suppression of axonal plasticity (206). M_1_R antagonism in STZ-induced T1DM mice and T2DM db/db mice through pirenzepine improved epidermal innervation, thermal hypoalgesia and slowing of nerve conduction velocities (206). M_1_R antagonists activate AMPK-PGC-1α pathway, enhancing mitochondrial energy production, sensory axon regeneration and axon function. M_1_R antagonism also corrected abnormalities of respiratory chain complex I and IV in the DRGs of diabetic mice, restoring their bioenergetic properties. Oxybutynin, a muscarinic antagonist approved in humans for bladder overactivity, promoted DRG neurite outgrowth *in vitro* in rat STZ T1DM sensory neurons. This was accompanied by enhanced mitochondrial basal and maximal oxygen consumption rate. Subcutaneous injection or topical application of oxybutynin to the hindpaw of T1 STZ-induced and T2 db/db diabetic mice improved thermal sensory loss, reversed allodynia in T1 mice and in the T2 mice preserved epidermal and corneal innervation (145). Oxybutynin, unlike pirenzepine, is a non-selective muscarinic antagonist. The neuroprotective effects offered by M_1_R antagonism were not restricted to DPN but also extend to other peripheral neuropathies such as chemotherapy and HIV-induced peripheral neuropathy (206).

There are several ongoing clinical trials evaluating the efficacy of pirenzepine in treating peripheral neuropathies, namely DPN and chemotherapy induced peripheral neuropathy. A 24-week DPN phase 2A clinical trial study demonstrated that daily topical 4% pirenzepine on the lower legs significantly increased epidermal innervation identified on skin punch biopsies from the lower limb (146). T2DM human subjects receiving daily topical 3% oxybutynin treatment for 6 months demonstrated an increase in epidermal axon density in leg skin biopsies and improved clinical pain and quality of life neuropathy scores (145). Given the collective findings to date, inhibition of M_1_R, may represent a therapeutic approach in treating DPN.

### Axon protection

Protection of axons in DM is an alternative approach that is newly emerging in clinical trials and might be applicable toward prevention of DPN. Several interventions may be possible to interrupt the complex cascade of axonal degeneration. Among the most important is that involving Sterile Alpha and Toll/Interleukin Receptor Motif-Containing Protein 1 (Sarm1), a critical mediator of axonal degeneration. Sarm1 functions as a metabolic sensor, responding to an increase in Nicotinamide Mononucleotide (NMN) to NAD^+^ ratio by cleaving residual NAD^+^, thereby leading to axon degeneration (153). Sarm1 deletion in rats was associated with remarkable preservation of distal axons 2 weeks after sciatic nerve transection injury. In control rats, axons degenerated within 24 hours (207). Furthermore, mice lacking the Sarm1 gene also exhibited resistance to distal axonal degeneration in a chemotherapy induced peripheral neuropathy model and a high fat diet-induced neuropathy model (152). In chronic (25 week) STZ-induced diabetic mice, Sarm1 knockdown prevented mechanical hypoalgesia and attenuated epidermal axon loss in the footpad skin. Sarm1 knockout was reported to support DM neurite outgrowth length, but this assay, as discussed further below in this chapter, remains problematic given insufficient evidence that DM impacts neuron growth *ex vivo*. Knockout suppressed sciatic nerve gene expression profiles associated with DPN, including Pvalb (parvalbumin), Cox (cyclooxygenase)7a1 and Cox 6a2 while elevating transcript levels of Lp1 (lipid transfer protein 1) (208). These specific ‘hits’ were generated by being classified with ‘neurodegenerative’ disease, but individually are of unclear significance.

The regulation of Sarm1 intracellularly has been recently explored. As a result of acute hyperglycemia *in vitro*, Sirtuin 3 (Sirt3) promotes deacetylation of Sarm1, enhancing its NAD^+^ cleavage activity, an outcome that exacerbates axon breakdown. Sarm1 deacetylation in response to high glucose levels in HT22 (immortalized hippocampal neurons) cells was only partially reversed by Sirt3 siRNA knockdown or the addition of Sirt3-H248Y, a catalytically inactive mutant form of Sirt3. Overall, these findings indicated that Sirt3, and perhaps other Sirt variants appear to act upstream of Sarm1’s NAD^+^ catalytic activity. Sarm1 was localized to mitochondrial membranes (209). Sarm1 deleted mice with superimposed mild T2DM (high-fat feeding combined with STZ), were transfected by AAV (adeno-associated virus vector) with an inactive mutant form of Sarm1 K641Q or wild type Sarm1. As expected, there was retained acetylation/inactivation of Sarm1 at K641 using the mutant construct. These mice treated with the mutant had mitigated features of DPN including thermal and mechanical sensory loss, epidermal denervation and a purported growth impairment of DRG sensory neurons. However, the latter *in vitro* assay, as above, remains an uncertain index of polyneuropathy since it is currently unclear whether chronic DM neurons have growth impairment *ex vivo*. Motor and sensory conduction recordings were not reported and the duration of the DM models was relatively short, at approximately 8 weeks. Nonetheless, there was attenuated ROS accumulation and restored ATP and NAD^+^ levels. Sirt3 null mice also had protection from the above features. In Sirt3 deleted mice treated with wild type or mutant K641 Sarm1 added by AAV (adeno-associated virus vector), there was exacerbation of DPN with wild type transduction but protection with the mutant Sarm1 (209). Overall, the Sirt3-Sarm1 axis may offer neuroprotection against axonal loss in DPN. The above mechanism however, does not explain the selective distal terminal loss of axons that characterizes DPN, but a potential route for enhanced axonal degeneration generally. Currently, there are at least two Sarm1 inhibitor listed drugs in clinical trials but they are for other indications.

Two additional targets may be important in axon protection. DUSPs, specifically types 1 and 4 (dual specificity phosphatases) are neuroprotective molecules expressed in DRG sensory neurons that may act upstream of Sarm1. DUSP1 and 4 upregulation was identified in a microarray screen of T1DM (STZ) long term mouse DRGs (210). It is possible that DUSP upregulation might counterbalance the impacts of heightened Sarm1 activation in diabetes suggested by the above work. The second molecule of interest is caspase-6, a facilitator of axon degeneration, likely downstream from Sarm1. Capase-6 was expressed and active in axons distal to injury and its inhibition delayed axon breakdown (156). Its role in DPN has not yet been evaluated.

### Epigenetic and genetic avenues

The potential roles of epigenetic regulatory RNAs have assumed increasing importance in understanding DPN. Both long non-coding RNAs (lncRNAs) and miRNAs can act as switches that turn specific genes “on” and “off” (211). In a survey of differentially regulated miRNAs in DRGs of chronic type 1 DM mice, two examples were analyzed, namely a 39% downregulation of mmu-let-7i and a 255% increase in mmu-miR-341 (212). Both were identified in sensory neurons and had impacts on neurite outgrowth and experimental DPN in mice. Intranasal delivery of a let-7i mimic improved neurite outgrowth *in vitro* and several features of DPN in type 1 DM mice including conduction velocities, sensation and epidermal innervation. An antimir, also given intranasally inhibiting miR-341 improved thermal sensation and conduction velocities. Knockdown of a lncRNA, Malat-1 (Metastasis Associated Lung Adenocarcinoma Transcript 1), upregulated in diabetic neurons, exacerbated features of experimental DPN (213).

Epigenetic mechanisms also involve the acetylation and methylation of histone proteins. During axon regeneration, these may involve RAGs (regeneration associated genes). For example, acetylation of p53, a tumor suppressor protein at K320 position by histone acetyltransferases (HATs), upregulated the expression of the RAGs Coronin-1b (an actin binding protein, impacts growth cones) and Rab1 (Ras-associated binding protein 1, regulates intracellular traffic), and promoted neurite outgrowth in cultured rat cortical neurons (214, 215). Alternatively, histone deacetylases (HDACs), act to oppose acetyltransferases. For example, inhibiting expression or pharmacological blockade of HDAC6 enhanced neurite outgrowth in cultured rat cortical neurons on inhibitory substrates (216). Future studies delineating the epigenetic regulation of the regenerative transcriptome in DPN are critical in learning how the regenerative capacity can be amplified to improve nerve regrowth in DPN.

More direct genetic approaches involving the transduction of plasmids or viral vectors, could involve delivery of growth factor proteins into muscles or tissues to protect from or reverse DPN (217). AAV-based gene delivery of NGF and BDNF into the fatty tissues of obese, diabetic mice with peripheral neuropathy (BTBR*^ob/ob^*) increased adipose nerve innervation as early as 2 weeks after treatment (218). Clinical trials have been initiated to deliver HGF into calf muscles through a plasmid vector to treat painful DPN with benefits demonstrated lasting up to 9–12 months (219). An important caveat, as discussed above, is whether associated axon sprouting would improve or exacerbate pain. Although a phase II trial identified apparent improvement of sensory neurological examination in DPN patients after 6-months of treatment with recombinant human nerve growth factor, a followup phase III trial was negative (220, 221).

### Overall translational challenges

While the identification and preliminary analysis of potential new molecular DPN targets is exciting, there are important barriers not yet surmounted. There is a challenging balance between growth and resilience to offset a variety of neuronal insults requiring a widely acting input, with potential physiological or detrimental side effects. This was described above in consideration of PTEN and Rb1 knockdown but likely applies to several other targets outlined such as APC, Rac1 and others. Interestingly, even insulin itself has raised concerns over its impact on oncogenesis. Direct exploitation of these pathways will require selective targeting over limited time periods. In the case of PTEN, it might be appropriate to simply shift PTEN levels back down to values identified in nondiabetics. Another possibility is that we may next be able to encounter a range of downstream mediators of growth beyond the above that are more selective in their roles. How to get siRNAs into the nervous system, for example to DRGs, is another challenge and may require novel approaches using stabilized or protected versions, or access through new routes such as intranasal administration, a route that offers insulin signaling to DRGs, (67). Another route is local dosing at the site of the growth cones, as discussed above. Other targets such as Sarm1 may have off target actions not yet clarified. The duration of treatment required also requires careful considerations. These include whether the approach is one of long term prevention or instead rescue and limitation of exposure. Finally, much of the preclinical work done in rodents requires careful pharmacodynamic metrics and clarification over whether human neurons would share these target properties.

## Current DPN therapy

Current DPN therapy, only briefly reviewed here, is essentially inadequate and largely unavailable excepting nonspecific pain interventions. Intensive glycemic control is effective for the primary prevention and secondary intervention of neuropathy in persons with T1DM (34). However, its benefits in neuropathy of T2DM is limited (222). Findings of glycemic control in T2DM suggest that there may be more complex management decisions based on specific mechanisms of neuropathy in T2DM.

For pain, previous and recently updated practice guidelines of the American Academy of Neurology (AAN guidelines) are relevant (223–225). Opioids are not routinely recommended. Gabapentinoids including pregabalin or gabapentin, which are calcium channel α2-δ subunit ligands, remain primary candidates. Serotonin-norepinephrine reuptake inhibitors (SNRIs), such as duloxetine also have medium level evidence and may help with coexisting depression. Older tricyclic antidepressant agents with similar actions including amitriptyline and nortriptyline have been of benefit but have additional, especially anticholinergic and cognitive side effects. Sodium channel inhibitors such as phenytoin, carbamazepine and other anti-epileptic agents have had some evidence of efficacy. At this time, topical therapy such as lidocaine and capsaicin patches are second line.

We have not reviewed additional non-pharmacological approaches to DPN that have varying levels of evidence to support their benefits. However physical exercise, in addition to its benefits from weight loss, has been shown to improve features of neuropathy (226). In the Italian Diabetes and Exercise Study, a 12-month supervised exercise program significantly improved multiple cardiometabolic risk factors, including HbA1c, systolic and diastolic blood pressure, low-density lipoprotein cholesterol (LDL-C), insulin resistance, and inflammatory markers, changes that were associated with a reduced risk of diabetic complications (227). In particular, a treadmill exercise program over 4 years in T1 and T2DM persons prevented declines in conduction and vibration sensation (228). Further, a resistance program of weekly exercise improved IENFD in the lower limb of persons with T2DM without clinical features of neuropathy (229). Resistance exercise can improve neuromuscular function (230), and reduce neuropathic pain in diabetic individuals (231). In addition to metabolic benefits, impacts on angiogenesis (232) and mitochondrial biogenesis (233, 234) and function as well as other mechanisms have been linked to exercise.

## Conclusions

There are still no effective treatments for arresting or reversing DPN. This relates to the complex pathogenesis of DPN that involves varying combinations of hyperglycemia, inflammation, mitochondrial dysfunction, oxidative stress, altered insulin signaling and dyslipidemia, and likely other direct impacts on neuron and SC molecular pathways. Targeting of single mechanisms may not be feasible or effective. Once loss of distal sensory terminals develops, it may be that attention toward specific regenerative approaches is required. These may differ from employing classical RAGs that are associated with acute injury and recovery outside of the DM context. Axon loss develops slowly in DPN, and mild symptoms may be unnoticed or dismissed for long periods. However, a range of new ideas targeting neuron function and growth cones are promising directions, discussed in this review. However, many of the studies to date have largely focused on nondiabetic injury models and are only preclinical. Nonetheless, new clinical trials may emerge from among this variety of potential targets, supported by ongoing preclinical model studies. These studies should continue to employ long term models, in order to be relevant to human disease. While many of the considerations around DPN are unique, findings identified in this line of work may be beneficial for translation to other neurological diseases.
